# Combination Immune Therapies to Enhance Anti-Tumor Responses by NK Cells

**DOI:** 10.3389/fimmu.2013.00481

**Published:** 2013-12-23

**Authors:** Ashley Mentlik James, Adam D. Cohen, Kerry S. Campbell

**Affiliations:** ^1^Immune Cell Development and Host Defense Program, The Research Institute at Fox Chase Cancer Center, Philadelphia, PA, USA; ^2^Abramson Cancer Center at the University of Pennsylvania, Philadelphia, PA, USA

**Keywords:** NK cells, immunotherapy of cancer, antibodies, monoclonal, ADCC, multiple myeloma

## Abstract

Natural killer (NK) cells are critical innate immune lymphocytes capable of destroying virally infected or cancerous cells through targeted cytotoxicity and further assisting in the immune response by releasing inflammatory cytokines. NK cells are thought to contribute to the process of tumor killing by certain therapeutic monoclonal antibodies (mAb) by directing antibody-dependent cellular cytotoxicity (ADCC) through FcγRIIIA (CD16). Numerous therapeutic mAb have been developed that target distinct cancer-specific cell markers and may direct NK cell-mediated ADCC. Recent therapeutic approaches have combined some of these cancer-specific mAb with additional strategies to optimize NK cell cytotoxicity. These include agonistic mAb targeting NK cell activating receptors and mAbs blocking NK cell inhibitory receptors to enhance NK cell functions. Furthermore, several drugs that can potentiate NK cell cytotoxicity through other mechanisms are being used in combination with therapeutic mAb. In this review, we examine the mechanisms employed by several promising agents used in combination therapies that enhance natural or Ab-dependent cytotoxicity of cancer cells by NK cells, with a focus on treatments for leukemia and multiple myeloma.

## Introduction

Natural killer (NK) cells are generally known for the ability to mediate spontaneous “natural” cytotoxicity of major histocompatibility complex class I (MHC-I)-deficient tumor or virus-infected cells. NK cells kill target cells through the release of perforin and granzymes from cytolytic granules or the surface expression of Fas ligand or TNF-related apoptosis-inducing ligand (TRAIL). Activated NK cells are also a potent source of interferon (IFN)-γ, tumor necrosis factor (TNF)-α, and a variety of other cytokines and chemokines that contribute significantly to early immune responses.

The activation of NK cells is controlled by a balance of signals emanating from a collection of germline-encoded activating and inhibitory receptors. Several inhibitory receptors, including CD94/NKG2A, members of the highly polymorphic killer cell-Ig-like receptor (KIR) family and ILT2/LIR-1/CD85j, play critical roles in tolerizing NK cells toward healthy cells by binding to MHC-I ligands [human leukocyte antigen (HLA)-A, -B, and -C allotypes] expressed on virtually all healthy cells (1). When NK cells engage with MHC-I-expressing healthy cells, the inhibitory receptors transduce negative signaling that efficiently abrogate stimulatory signals from co-engaged activating receptors at the immunological synapse (2–4). Many tumors and virus-infected cells down-regulate their surface expression of MHC-I to avoid recognition by the antigen receptor on cytotoxic T cells, but these abnormal cells inherently become susceptible to NK cell-mediated attack in the absence of the tolerizing MHC-I ligands.

Activating receptors expressed on human NK cells include FcγRIIIA (CD16), activating forms of KIR, 2B4, NKG2D, and the natural cytotoxicity receptors (NCR), which are also known as NKp30, NKp44, and NKp46 (5, 6). Of these, the NCR and NKG2D are particularly important receptors for triggering NK cell responses toward tumor target cells (7). In contrast to inhibitory receptors, triggering of the activating receptor NKG2D is a key mechanism by which NK cells recognize stressed or diseased cells and destroy them (8). NKG2D specifically recognizes MHC chain-related (MIC) A, MICB, and UL16-binding proteins (ULBPs) in humans, which are HLA-related molecules lacking peptide presentation capacity that are not expressed on normal cells but are up-regulated on stressed cells, such as tumors (8, 9).

Thus, NK cells are important effectors in immune responses to tumors and viral infections, and increased understanding of the mechanisms controlling NK cell activation has led to the development of therapeutic agents that can improve their responsiveness. While these agents show promise, results are inconsistent between patients due to inherent differences in activity/function of an individual’s immune system, and expression of distinct biomarkers on cancers that can differentially influence NK cell responsiveness. A growing variety of treatment options can improve outcome for individual patients. Novel treatment regimens combining new and old therapies are even showing promise among relapsed and refractory patients. Here, we review some of the currently available therapies that are known to stimulate NK cell functions and how they are being used in combination with other agents to boost anti-tumor responses in cancer patients.

## Antibody-Dependent Cellular Cytotoxicity

FcγRIIIA (CD16) provides NK cells with the capacity to mediate antibody-dependent cellular cytotoxicity (ADCC) upon recognition of the Fc segment of IgG bound to cell surfaces. This antigen-specific NK cell targeting mechanism appears to play an important role in tumor eradication by several therapeutic tumor-specific mAb, such as herceptin, rituximab, and elotuzumab. Interestingly, Deguine et al. recently suggested that NKG2D engagement might be crucial for NK cell-mediated ADCC responses. In that study, mouse tumors expressing NKG2D ligands induced enhanced ADCC degranulation, while responses were reduced toward tumors lacking NKG2D ligand. The results indicate that the mAb-FcR interaction mainly stabilized adhesion with the target cell to contribute contact stability, while NKG2D triggering provided the activation threshold needed to trigger NK cell degranulation (10). This concept is supported by earlier work in a mouse model of renal cancer that showed NKG2D ligation does not stimulate strong adhesion with tumor cells. In that report, target cells that did not express ICAM-1 were not killed in an NKG2D-dependent manner, but transfection of ICAM-1 into these cells restored NKG2D-mediated cytotoxicity (11).

An emerging strategy to enhance NK cell-mediated ADCC responsiveness is to inhibit the shedding of CD16 that occurs after NK cell activation (12, 13). Specifically, CD16 can be cleaved by metalloproteinases after being triggered by an IgG-opsinized target cell or treatment with IL-2, but if metalloproteinases are inhibited, CD16 is not shed. In fact, CD16 signaling is sustained and target cell killing and cytokine production are enhanced in the presence of metalloproteinase inhibitors (14). Furthermore, combining metalloproteinase inhibition with a bispecific antibody (against CD16 and CD33) resulted in a sustained and very specific anti-tumor response (15). Such a combination strategy has the potential to substantially increase NK cell responses to cancer.

An additional immunoglobulin-based strategy to target more immune responses toward tumor cells is to create bispecific or trispecific antibodies. The engineering of bispecific monoclonal antibodies (mAb) that create surrogate bridging between activating receptors on T or NK cells with tumor-specific antigens have shown therapeutic promise for decades (16). A common bispecific design theme to target NK cells has employed fusing antibody Fv domains that bind CD16 with Fv domains that bind tumor cell markers, such as CD30 (17), ErbB2 (18–20) or CD19 (21, 22). These constructs can trigger ADCC responses by NK cells and monocytes/macrophages through the CD16 FcR without the need for an incorporated Fc domain. As a cautionary tale for appropriate design, a bispecific incorporating a CD16-binding Fv domain in combination with a CD16-binding Fc domain was found in an early phase I study to induce toxicity through dimeric engagement of the FcR on the surface of monocytes and NK cells in the blood, which induced rapid release of cytokines, including TNF-α, IL-6, and IL-8 (18). Modern recombinant approaches fuse Fv domains into single chain constructs that have been termed bispecific or trispecific killer cell engagers (BiKEs or TriKEs), which lack Fc domains entirely. Importantly, these BiKEs and TriKEs can be highly effective at inducing NK cell ADCC and cytokine responses (22). In another approach, von Strandmann et al. created a bispecific protein (ULBP-BB4) that fused the NKG2D ligand, ULBP2, onto a single chain Fv targeting CD138, which is highly expressed on a number of malignancies, including multiple myeloma (MM). In xenograft mouse models injected with human tumor cells and human PBLs, ULBP-BB4 demonstrated potent anti-tumor activity while not significantly harming healthy cells (23). Many researchers have also developed trivalent antibodies composed of two tumor antigen-recognition domains and one monovalent domain that recognizes NK cells (19–21, 24). If properly manipulated for safety, bispecific and trispecific antibodies offer a very targeted approach to effective tumor therapy that can directly involve NK cell effector function.

## The Use of NK Cell-Related Immune Therapies to Treat Multiple Myeloma

A common proving ground for testing immune therapies that stimulate NK cell activity has been MM. MM is a deadly hematologic cancer characterized by clonal expansion of malignant plasma cells that reside in the bone marrow and thrive on interactions with the bone marrow microenvironment (25). Despite advances in treatment strategies, MM remains an incurable disease, with about 20,000 newly diagnosed cases and over 10,000 deaths per year in the U.S. (26). Novel therapies have improved survival over the last decade, including autologous hematopoietic stem cell transplantation (HSCT) and the use of new drugs (27). Allogeneic stem cell transplantation can be curative, but is often associated with high transplantation-related mortality (28). Despite these advanced therapeutic options, median survival remains around 4–5 years in adults (29) and the development of better treatments is essential. Interestingly, evidence is accumulating that NK cells may play a prominent role in immune responses toward MM and can also contribute to graft-versus-myeloma responses in haploidentical HSCT (30), It is becoming clear that NK cells can elicit potent allogeneic and autologous responses to myeloma cells *in vitro* and in patients (30, 31). Given the importance of NK cells in immune responses toward MM, combination therapies that enhance NK cell functions are showing promise in treating this deadly disease, as will become evident in the following discussion.

## Immunomodulatory Drugs (IMiDs®)

Thalidomide, lenalidomide, and pomalidomide form a new class of immunomodulatory drugs, referred to as IMiDs, which can broadly stimulate the functions of NK cells and T cells to treat cancer (32). Thalidomide is a glutamic acid derivative with a dark history as a therapeutic agent, since it caused severe birth defects when used to treat morning sickness in pregnant women in the late 1950s. Nonetheless, it was subsequently found to have anti-inflammatory, anti-angiogenic, anti-proliferative, and immunomodulatory properties that fostered further investigation (33–35). The anti-inflammatory properties of thalidomide are at least partially due to potent inhibition of the production of TNF-α by activated monocytes (35). Lenalidomide and pomalidomide are more potent thalidomide analogs that have since emerged (36), and pomalidomide is even more potent at co-stimulating T cells than lenalidomide (37). Since these IMiDs can enhance the functions of T cells and NK cells, suppress angiogenesis, inhibit TNF-α production, and directly repress tumor cell growth, they are potentially beneficial in treating cancer. To date, both lenalidomide and pomalidomide have been used to treat MM and a variety of other cancers.

The mechanism of immune stimulation by IMiDs is complex and not entirely established (32). Treatment of patients with lenalidomide has been shown to increase the overall frequency of NK cells in peripheral blood, suggesting that they either proliferate or migrate into the bloodstream (38–40). Lenalidomide does not appear to stimulate NK cells directly, however, but instead functions through effects on other leukocytes in peripheral blood (40). Stimulation of T cells by lenalidomide overcomes the need for signals from antigen presenting cells and induces increased proliferation and enhanced production of the type 1 cytokines, IL-2, and IFN-γ (37, 41, 42). At least part of the stimulatory effects of IMiDs on NK cells appears to be due to the T cell production of IL-2, which is a potent growth factor for NK cells (43, 44). Both lenalidomide and pomalidomide have also been shown to increase ADCC activity by NK cells (44, 45). At least part of this effect may result from an increased frequency of the CD56^dim^ NK cells expressing CD16 and LFA-1 in peripheral blood, which are responsible for mediating ADCC (46). This ability of IMiDs to augment ADCC has been borne out in clinical studies, particularly in combination with the CD20-targeting antibody rituximab, where significant activity has been seen in relapsed/refractory B-cell lymphomas and chronic lymphocytic leukemia (47, 48). In MM, lenalidomide is usually used in combination with steroids (49, 50). However, the enhanced NK cell-mediated responses by lenalidomide can be reversed in combination with dexamethasone (40), suggesting that using steroids long-term in combination with lenalidomide may be counterproductive to its immune-stimulatory effects, and that steroid-free combinations should be explored. It should also be noted that tumor cell lines cultured in lenalidomide become more susceptible to NK cell-mediated lysis, due to their increased expression of ligands for NK cell activating receptors (38–40, 51). Taken together, NK cell-mediated anti-tumor responses can be stimulated in a variety of ways by IMiDs, and this enhanced function can be beneficial in treating cancer.

## Bortezomib

Bortezomib is an inhibitor of the 26S proteasome that is currently used to treat MM and lymphoma. Inhibition of the proteasome has several direct negative impacts on tumor cells, including inhibiting proliferation and inducing apoptosis, but bortezomib-treated tumor cells also become more susceptible to attack by NK cells (52). Upon inhibition of the proteasome, tumor cells are incapable of processing and presenting proteolytic peptide fragments on MHC-I molecules on the plasma membrane. Consequently, bortezomib down-regulates the surface expression of MHC-I on tumor cells *in vitro* and *in vivo* (53), thereby reducing the levels of this important protein for NK cell tolerance and enhancing susceptibility to NK cell-mediated natural and antibody-dependent cytotoxicity (54, 55). Bortezomib treatment can augment the efficacy of adoptively transferred NK cells in murine tumor models (56), and this approach has now been translated to the clinic for cancer patients (57).

## Elotuzumab

A promising new monoclonal antibody candidate for treatment of MM is elotuzumab (formerly HuLuc63). Elotuzumab is a fully humanized antibody that recognizes the SLAM family member CS1 (CRACC, SLAMF7, CD319), a surface glycoprotein normally expressed on NK cells, monocytes, mature dendritic cells, a subset of T cells, and stimulated B cells (58, 59). Normal plasma cells express high levels of CS1, which correlates with high expression on MM cells (60). CS1 is an attractive therapeutic ADCC target because the available clinical data indicate that expression persists on MM cells even after conventional treatments (61–63). CS1 was originally found to engage in homotypic interactions as a self-ligand, and pretreatment of a NK cell line with recombinant CS1-Ig fusion protein was shown to stimulate killing of K562 target cells, apparently by directly activating the NK cells via homotypic interaction (64). Two separate reports found high CS1 expression in most MM cases studied, and elotuzumab was found to significantly increase NK cell-mediated ADCC of primary MM cells (65, 66).

While initiation of NK cell-mediated ADCC upon engagement with CD16 is the best characterized function of elotuzumab, the exact mechanism(s) of action is unclear (67). Importantly, CS1 is also considered a co-stimulatory receptor on NK cells (64, 68), and recent evidence indicates that elotuzumab may also stimulate NK cells directly through direct interactions with CS1 on the NK cell surface (69). As an additional potential mechanism, elotuzumab may interfere with interactions of MM cells with the bone marrow microenvironment, which is a key requirement for tumor survival and proliferation (60, 67). MM cell interactions with bone marrow stromal cells, osteoclasts, and osteoblasts lead to bone deterioration, angiogenesis, and MM cell survival and proliferation (70), so disrupting the ability of MM cells to interact with the microenvironment could provide multiple benefits. All of these potential mechanisms of action continue to be explored, though so far the data continue to indicate that NK cells contribute to the therapeutic efficacy of this anti-CS1 monoclonal antibody.

Clinically, elotuzumab used alone was well-tolerated and led to disease stabilization in a subset of relapsed/refractory myeloma patients (63). More promising clinical activity was seen, however, when it was used in combination with lenalidomide and dexamethasone, with over 80% of relapsed patients responding in phase I and II trials, and progression-free survival significantly longer (median 26.9 months at the 10 mg/kg dose) than that previously observed for lenalidomide/dexamethasone alone (62). Elotuzumab-mediated ADCC of MM targets by NK cells can be enhanced *in vitro* by pretreatment with a proteasome inhibitor (54, 66), and a small combination study of elotuzumab and bortezomib in relapsed/refractory myeloma patients showed this combination to be safe, with preliminary efficacy observed (61). Currently, three randomized phase III trials are underway adding elotuzumab to either bortezomib or lenalidomide/dexamethasone, in both newly diagnosed and relapsed/refractory MM patients. These studies will more definitively assess if there is a benefit to adding elotuzumab to these standard therapies.

## KIR-Blocking Monoclonal Antibody

Allogeneic HSCT has emerged as an effective treatment option for a variety of hematological cancers after chemotherapeutic ablation of the recipient’s immune cells (71). Because of the high polymorphic variability of KIR and MHC-I in the human population, variability of these receptor/ligand pairs should be considered in the context of transplantation. Velardi and colleagues first reported that donor allogenicity of NK cells in HSCT to treat acute myeloid leukemia (AML) resulted in a double benefit by enhancing anti-leukemia responses, while reducing graft-versus-host disease (72). In the absence of an HLA-identical sibling donor, haploidentical HSCT is commonly used, in which a mismatch exists in at least one HLA allele. This mismatch improves the odds that at least one inhibitory KIR in the donor NK cells lacks an MHC-I ligand in the transplant recipient. In this scenario, the donor-derived NK cells are less inhibited and considered to be more capable of triggering a graft-versus-leukemia effect. KIR/HLA mismatch in HSCT has resulted in improved outcomes by several groups, specifically in treating AML (73–75), and may play a role in myeloma as well (30, 76). It is believed that NK cell-mediated autoimmunity does not occur in these patients because healthy cells are less likely to up-regulate the stress ligands that trigger NK cells (77). It is important to note that recent evidence indicates that donors expressing activating KIR (especially donors expressing KIR2DS1, but lacking its ligand, HLA-C2) also contribute significantly to improved outcomes in HSCT to treat AML (78, 79). These results indicate that certain engineered mismatches of KIR/HLA interactions in the context of HSCT can significantly influence NK cell responses in AML patients and perhaps other cancers.

In addition to exploiting KIR/HLA ligand mismatch in the context of HSCT, monoclonal antibody-mediated blockade of the KIR/HLA interaction has emerged as a potential cancer immunotherapy to lower the threshold of NK cell activation. The *in vitro* use of mAb to block KIR function was first found to increase cytokine production in T cells in 1996 (2). To further test this concept *in vitro*, Binyamin et al. potentiated NK cell responses to autologous EBV-transformed B cells by combining a panel of mAbs to block numerous NK cell inhibitory receptors (KIR, CD94/NKG2A, and ILT2/LIR-1/CD85j) in combination with the B cell-specific anti-CD20 mAb rituximab to simultaneously reduce inhibitory signals and trigger ADCC, respectively (80). Importantly, NK cell-mediated cytotoxicity of the transformed target cells was not elevated by inhibitory receptor blockade alone, suggesting that other tolerizing mechanisms effectively prevent the attack of normal cells in the context of these inhibitory receptor-blocking conditions.

Based on this concept, a humanized KIR-blocking mAb IPH2101 (formerly 1-7F9) has been developed and is currently being tested in clinical trials. IPH2101 is a pan-specific anti-KIR antibody that binds KIR2DL1, -2 and -3, which are the most relevant inhibitory KIR family members, due to their combined capacity to recognize all allotypes of HLA-C. The antibody was shown to block the interaction between these inhibitory KIR2DL and HLA-C and also binds the activating receptors KIR2DS1 and KIR2DS2, although the functional impacts of these interactions have not been formally tested (77). *In vitro* preclinical studies demonstrated that IPH2101 mAb augments NK cell cytotoxicity of HLA-C-expressing tumor cells without targeting normal blood mononuclear cells, which is critical to assure that NK cells remain tolerant in treated patients (77). This was confirmed in a preclinical mouse model that was engineered to co-express KIR2DL3 and its ligand, HLA-Cw3, which was able to license or educate the mouse NK cells. When KIR was then blocked *in vivo* with IPH2101, the mouse NK cells were able to destroy HLA-Cw3-positive target cells without development of autoimmunity (81).

In view of the capacity of NK cells to respond to myeloma cells, phase I clinical trials have been initiated to treat MM patients with IPH2101 and the modified variant, IPH2102. When used alone in patients, the side effects of IPH2101 were minimal with no evidence of autoimmunity, and *ex vivo* functional assays showed enhanced NK cell cytotoxicity (82, 83). A trial of IPH2101 in combination with lenalidomide has since been initiated, based upon a variety of effects by these reagents that can potentially synergize to enhance NK cell responses. IPH2101 is expected to enhance NK cell killing by blocking inhibitory signals, while lenalidomide can stimulate general NK cell function and may even up-regulate triggering ligands on MM cells (51).

Importantly, the addition of KIR blocking mAbs may prove to be an asset in treating a variety of cancers, as a way to boost the potential of NK cells to kill stressed or cancerous cells, while retaining general NK cell tolerance toward normal cells. Nonetheless, it is possible that optimal clinical responses may require a combination therapy of multiple antibodies blocking a wider variety of inhibitory receptors expressed on NK cells, such as KIR3DL family members, CD94/NKG2A, and ILT2/LIR-1/CD85j, as well as the addition of an ADCC targeting mAb to stimulate tumor-specific cytotoxicity, as demonstrated by the *in vitro* studies of Binyamin et al. described earlier (80).

## Agents Promoting the Expression of NKG2D Ligands on Tumor Cells

The human NKG2D ligands MICA, MICB, and ULBPs are commonly up-regulated on stressed or infected cells and thereby provide a key recognition element for NK cell-mediated attack of many cancer cells (8, 9). Several cancer types are able to shed NKG2D ligands into the sera as an immune evasive mechanism, and these soluble ligands have been shown to cause down-regulation of NKG2D on NK cells, which leads to a stunted immune response (84–87). Demonstrating the high frequency of shed ligands, Hilpert et al. recently found at least one soluble NKG2D ligand in the sera of 183 leukemia patients analyzed, and culture of NK cells in leukemia patient sera resulted in down-regulation of NKG2D expression (88). Shedding of the NKG2D ligand, MICA, by chronic lymphocytic leukemia cells can be induced upon translocation of the endoplasmic reticulum-resident proteins ERp5 and GRP78 to the tumor cell surface (89). Additionally, shedding of MICA/B has been attributed to proteolytic activity of the ADAM10 and ADAM17 metalloproteinases in some tumor cell lines (90). The MICA*008 allele can also be released into exosomes, which can subsequently down-regulate NKG2D expression and reduce NK cell-mediated cytotoxicity (90, 91). On the other hand, the expression of CEACAM1 in cancer cells can cause the intracellular retention of NKG2D ligands, thereby limiting NK cell detection through NKG2D (92). These observations have made the retention/upregulation of NKG2D ligands on tumor cells an attractive goal for cancer therapy.

Many drugs that were first considered for cancer therapy because they can alter gene expression in tumor cells were subsequently found to also increase the susceptibility of tumor cells to cytotoxicity by NKG2D-expressing NK cells. These drugs include those that promote gene upregulation (93–96), differentiation (97–99), and DNA or protein damage (100–103). For example, HDAC inhibitors cause the upregulation of NKG2D ligands, MICA/B and ULBPs, in tumor cells but not healthy cells. Treatment with the HDAC inhibitor valproic acid (VPA, valproate) leads to higher expression of NKG2D ligands at the transcriptional and translational levels by inducing acetylation of the histones bound to MICA and MICB gene promoters (104, 105). The relatively low toxicity and low occurrence of off target effects of VPA make it a reasonable means of boosting effector cell function. Furthermore, treating cultured cells with VPA and the DNA methylation inhibitor hydralazine was shown to increase dimethylated MICA/B gene promoters, thereby further stimulating transcription (106, 107). The addition of a metalloproteinase inhibitor has also been shown to block the proteolytic cleavage of NKG2D ligands, as a means to further decrease shedding into the sera when used in conjunction with VPA treatment (108). Lastly, spironolactone, a diuretic commonly used to treat heart failure and high blood pressure, has very recently been found to also upregulate NKG2D ligands and therefore increase NK cell cytotoxicity of colon cancer cell lines (109).

Glycogen synthase kinase (GSK)-3 has recently been discovered as a new target to promote MICA/B upregulation. GSK3 inhibitors have been widely used to suppress the proliferation of malignant lymphoid cells, but Fionda et al. recently showed that GSK3 inhibition also increased MICA expression at the protein and mRNA levels in MM cells (110). In conjunction with the increased MICA expression, the addition of the GSK3 inhibitor significantly enhanced NK cell-mediated cytotoxicity of tumor cells. Mechanistically, GSK3 inhibition correlated with the down-regulation of STAT3, which is a negative regulator of MICA expression (110). In addition, the combination of lenalidomide with GSK3 inhibition further enhanced MICA expression, which further supports the combined mechanistic benefit of these agents with current anti-tumor therapies.

## Anti-CD137 Monoclonal Antibodies

CD137 (4-1BB, TNFRSF9) is an inducible member of the TNF receptor superfamily that functions as a co-stimulatory signaling molecule on the surface of activated T and NK cells. CD137 ligation further augments activation of these cells, increasing their proliferation, cytokine secretion, and effector function, and preventing activation-induced cell death (111, 112). Treatment with agonist anti-CD137 mAb can mimic this co-stimulatory signal, leading to regression of large tumors in multiple murine models, including B-cell lymphoma and myeloma (111, 113–115). This effect requires CD8^+^ T cells, but is also dependent upon the presence of NK cells (116, 117), implying an impact on these cells as well. Many diverse types of tumors express elevated levels of the CD137 ligand, CD137L, accompanied by increased expression of CD137 on immune cells within the tumor environment, while expression of either is negligible on healthy cells (115, 118–120).

There are conflicting data regarding the effects of anti-CD137 mAb on NK cells. CD137 engagement on mouse NK cells consistently results in activation, but this can be either activating or inhibitory in humans, depending on the setting or model used. In human leukemia cells, the CD137/CD137L interaction can also result in bidirectional signaling to suppress NK cell-mediated responses; CD137 recognition of CD137L on leukemic cells transmits inhibitory signals into the NK cell to impair cytokine production and cytotoxicity responses and CD137L engagement by CD137 induces the production of TNF and immunosuppressive interleukin (IL)-10 by the tumor cells (119). Inhibiting this interaction using soluble CD137-Fc or a “blocking” anti-CD137 mAb can restore NK cell cytotoxicity (119). Taking this rationale one step further, Buechele et al. suggested the potential merits of a dual strategy of blocking CD137/CD137L interaction and neutralizing immunosuppressive TNF (121). Lin et al. demonstrated that human NK cells up-regulate CD137 *in vitro* following Fc-receptor engagement, which promotes release of pro-inflammatory cytokines but decreased cytotoxicity against K562 targets. This implies a negative impact of CD137 expression and signaling in human NK cells, though the direct impact of CD137 ligation was not tested in this model (122, 123). In contrast, Kohrt et al. have shown that following FcR-induced CD137 up-regulation on NK cells, adding an agonist anti-CD137 mAb known to induce CD137 signaling actually enhances NK cell-mediated ADCC toward rituximab- or trastuzumab-coated target cells (124, 125). Whether this antibody may also be working by preventing CD137 on immune cells from binding to CD137L and inducing “reverse” signaling within the tumor cell was not explored in these studies. Nonetheless, these data have led to an ongoing clinical trial (NCT01775631) combining rituximab with agonist anti-CD137 mAb (BMS-663513, urelumab) in patients with relapsed/refractory B-cell malignancies.

These inconsistent findings on the impacts of manipulating CD137 in NK cells are likely the result of signaling differences between mice and men, the use of different CD137-targeting reagents, and even between different hematopoietic versus non-hematopoietic cancer models (120). For clinical relevance, it will be important to better understand the human mechanistic impact of CD137/CD137L interaction in order to properly exploit the potential treatment options. The role of CD137/CD137L interactions on interplay between NK cells and CD4^+^, CD8^+^, and regulatory T cells (Tregs) is also incompletely understood (126). For instance, the depletion of immunosuppressive Tregs has been shown to enhance the anti-tumor activity of anti-CD137 mAb (115). However, CD137 engagement on CD4^+^ and CD8^+^ T cells is believed to always be activating in mice and men (126). Nonetheless, while the exact mechanism is still unclear, the addition of the CD137 mAb along with neutralization of immunosuppressive cells and signals shows potential to boost immune function when added to current cancer therapies (121, 127).

## Anti-GITR Monoclonal Antibodies

Glucocorticoid-induced TNF receptor (GITR, TNFRSF18) is another co-stimulatory member of the TNF receptor superfamily expressed on T cells, NK cells, and B cells, among other hematopoietic cell types (126). GITR expression is generally low in resting T and NK cells, is up-regulated after activation, and the receptor is expressed constitutively at high levels in CD4^+^Foxp3^+^ Tregs. GITR is an activating receptor in T cells, since *in vitro* or *in vivo* engagement with GITR ligand or agonist anti-GITR mAb has been reported to support the expansion of CD4^+^ and CD8^+^ T cells and renders T cells resistant to suppression by Tregs (128, 129). Treatment with agonist anti-GITR mAb leads to enhanced vaccine-induced and endogenous effector T cell responses and tumor rejection in multiple murine tumor models, and is associated with a marked reduction in Treg frequency within the tumor microenvironment, though the exact mechanism(s) by which GITR ligation modulates Tregs remains controversial (115, 130–133). Based on these preclinical findings, the first clinical trial of an agonist anti-human GITR mAb (TRX518) in patients with advanced melanoma or other solid tumors is now underway (NCT01239134).

Similar to CD137, there is conflicting evidence about whether GITR is activating or inhibitory in human NK cells, which may also reflect differential NK cell responses to GITR engagement between mice and men (120). While agonist anti-GITR mAb can augment murine NK cell proliferation and cytotoxicity, GITR engagement on human NK cells has been reported to block NF-κB activation, cause release of anti-inflammatory cytokines, suppress NK cell proliferation, and increase NK cell apoptosis (134). Furthermore, Baltz et al. found that soluble GITR ligand (sGITRL) is released by several hematologic malignancies, detectable in patient sera, and these patients display reduced NK cell cytotoxicity and IFN-γ production (135). In CLL, bidirectional GITR/GITRL signaling can support tumor cell growth by causing release of survival factors, such as IL-6, IL-8, and TNF, and interfering with rituximab-induced ADCC responses (136). However, an antagonistic anti-GITR mAb can block GITR-GITRL interaction, and restore NK cell-mediated ADCC responses. Finally, a dual strategy has been developed by Schmiedel et al. that has potential to enhance existing therapies for AML and CLL (137). An Fc-optimized GITR-Ig fusion protein was found to block the GITR/GITRL interaction and target GITRL-bearing cells for ADCC, thus enhancing NK cell-mediated cytotoxicity of cancer cells. Hence, like anti-CD137 mAb, anti-GITR mAb, and other GITR-targeting therapies have the potential to boost the effectiveness of current cancer therapies by virtue of pro-inflammatory and cytotoxic effects involving NK cells.

## PD-1 or PD-L1 Blocking Monoclonal Antibodies

Programed death 1 (PD-1; CD279) is a member of the B7 family of co-signaling receptor that is up-regulated on activated T cells, NK cells, B cells, dendritic cells, and monocytes (138). The intracellular domain of PD-1 contains an immunoreceptor tyrosine-based inhibitory motif (ITIM), which can recruit the protein tyrosine phosphatases SHP-1 and SHP-2 to mediate inhibitory signaling (139). The engagement of PD-1 by its ligands, PD-L1 or PD-L2, blocks the immune response in both T and NK cells by inhibiting PI3K/Akt and Ras activation signaling (140–142). PD-1 expression also marks “exhaustion” in T cells, and engagement of PD-1 can cause apoptosis of CD8^+^ T cells and the differentiation of CD4^+^ T cells into immunosuppressive Tregs (143–146). In NK cells, PD-1 engagement impairs activation, conjugate formation, cytotoxicity, and cytokine production (145, 147, 148). In healthy tissue, PD-L1-induced inhibitory signaling minimizes damage to bystander cells and prevents excessive immune responses during acute infections (149). However, many tumor and viral models express PD-1 ligands as an immune evasion mechanism. In contrast, PD-L1 is expressed at low levels on healthy tissue, and resting NK cells express low levels of PD-1 (144, 145, 147, 148). IFN-γ can potently up-regulate PD-L1 expression (150), suggesting that localized cytokine production by NK cells and Th1 cells may actually promote PD-1-based immune evasion by tumors.

The disruption of PD-1/PD-1 ligand interactions can significantly potentiate immune responses to viral infections and cancer, and antibody-mediated blockade of these interactions has emerged as a prime target for immune therapies. In the case of viral persistence in the liver, the abrogation of PD-L1 by siRNA was shown to enhance the number of intrahepatic NK cells and CTL, thereby increasing cytotoxicity, cytokine production, viral clearance, and memory (148). During HIV infection, PD-1 levels increase on patient NK cells, and this has been shown to diminish NK cell proliferation (145). PD-L1 expression on tumors and PD-1 expression on tumor-infiltrating lymphocytes have been associated with poor outcome in renal cell carcinoma patients (151, 152). We have recently reported significantly increased expression of PD-1 on cytolytic NK cells in renal cell carcinoma patients, suggesting that these tumors can directly suppress tumor-infiltrating NK cells by this mechanism (153). Remarkably, the PD-1 expression on NK cells and other leukocytes was rapidly reduced after surgical resection of the primary renal tumor. Anti-PD-1 or anti-PD-L1 mAbs block the interaction of PD-1 on T and NK cells with its ligand, PD-L1 and this restores the function of exhausted cytolytic T cells, augments T cell proliferation, and enhances NK cell cytokine production and cytotoxicity responses, leading to enhanced anti-tumor effector responses and tumor regression in multiple murine models (146, 149). In clinical trials of anti-PD-1 and anti-PD-L1 antibodies to treat a variety of solid tumors, objective, often durable responses were seen in up to a third of patients, demonstrating proof of principle for this approach, and patients that responded to the treatment were shown to express PD-L1 in their tumors (154, 155). Further clinical trials of several candidate antibodies in both solid and hematologic cancers are ongoing.

In studies of MM patients, Benson et al. found upregulation of PD-1 on NK cells in conjunction with PD-L1 expression on MM cells, and *in vitro* treatment with an anti-PD-1 mAb enhanced NK cell conjugation with PD-L1-expressing MM target cells, resulting in enhanced cytotoxicity and IFN-γ production (147). In the same study, *in vitro* treatment with lenalidomide was shown to further enhance NK cell responsiveness by lowering PD-L1 expression on MM cells. In a mouse model of MM, Hallett et al. also saw increased levels of PD-L1 on MM cells, along with an exhausted phenotype in T cells, release of immunosuppressive IL-10 and expansion of Treg cells accompanied by increased levels of PD-1 on T and NK cells (144). Blockade of the PD-1/PD-L1 interaction with a PD-L1-specific mAb increased mouse survival by 40%. Like CS1, PD-1 expression persists after stem cell transplant and prior treatment, so PD-1 is a reliable target in relapsed or refractory cancer. Although PD-1 upregulation on human NK cells has only been reported in MM and renal cell carcinoma to date, its expression may be elevated in a variety of cancers as a mechanism to suppress anti-tumor responses. Therefore, the addition of PD-1/PD-L1 blocking mAb to an existing treatment regimen shows encouraging promise in boosting anti-tumor and anti-viral responses by several immune cell types, including NK cells, through counteracting a potent mechanism of immune evasion.

## Conclusion

A variety of new therapeutic agents have recently become available to potentiate NK cell responses in cancer patients, and many of these drugs are either approved or undergoing clinical trials (see Table 1). Here we have discussed a representation of this expanding toolbox of agents that allows clinicians to potentiate NK cell functions and thereby enhance anti-tumor therapy. Major future goals will be to expand upon these therapeutic options, better define mechanisms of action for these agents, identify specific combination therapies that are most effective at boosting NK (and T) cell function, and identify biomarkers (e.g., PD-L1 expression on tumor cells) that may better predict which patients are most likely to respond to these immune therapies. Furthermore, while MM has served as an appropriate proving ground for testing the therapeutic efficacy of several of these agents (156), they will need to be tested on other cancers, as well as viral infections and other disease states, to expand their usefulness.

**Table 1:** **Summary of some major therapeutic agents discussed in the text**.

Agent	Examples	NK cell-specific mechanism	Disease studied	Reference
Immuno-modulating drugs (IMiDs)	Thalidomide, lenalidomide, pomalidomide	Stimulate NK and T cells to release cytokines, kill tumor cells, block angiogenesis	MM, leukemia, NHL, pancreatic, esophageal, prostate	Sampaio et al. (33), D’Amato et al. (34), (35) Hayashi et al. (44), Wu et al. (45), Quach et al. (36), clinicaltrials.gov
Proteasome inhibitors	Bortezomib	Induce tumor cell death, increase NKG2D ligand expression	MM, leukemia, lymphoma, hepatocellular	Armeanu et al. (160), Shi et al. (53), (55) Moreau (156), clinicaltrials.gov
Anti-CS1 antibody	Elotuzumab	Triggers NK cell ADCC	MM	Hsi et al. (65), Tai et al. (66), (54) Benson and Byrd (67), Moreau (156)
Anti-KIR2DL antibody	IPH2101, IPH2102 (Lirilumab)	Blocks inhibitory KIR	MM, leukemia	Binyamin et al. (80), Sola et al. (81), Benson et al. (51), Vey et al. (83), clinicaltrials.gov
HDAC inhibitors	Valproic acid, panobinostat, vorinostat	Increase NKG2D ligand expression	MM, leukemia, Hodgkin’s lymphoma, hepatocellular	Armeanu et al. (93), Yamanegi et al. (104), Moreau (156), Yang et al. (105), clinicaltrials.gov
Anti-CD137 antibodies	BMS-663513 (urelumab)	Augment NK cell ADCC, co-stimulatory in T cells	NHL, melanoma, breast cancer	Baessler et al. (119), Buechele et al. (121), Kohrt et al. (125), clinicaltrials.gov
Anti-GITR antibodies	TRX518	Block GITR/GITRL in NK cells, co-stimulatory in T cells, neutralize Tregs	Solid tumors, melanoma	clinicaltrials.gov
Anti-PD-1 antibodies	BMS-936558 (nivolumab), CT-011, MK-3475	Block PD-1 receptor, block PD-1/PD-L1 interaction in NK and T cells	Hepatitis C, renal, prostate, melanoma, MM, colorectal, NHL, NSCLC	Benson et al. (147), Brahmer et al. (154), Rosenblatt et al. (161), Gardiner et al. (162), Lipson et al. (163), clinicaltrials.gov
Anti-PD-L1 antibodies	BMS-936559, MSB0010718C, MPDL3280A	Block PD-L1 interaction with receptor PD-1 in NK and T cells	Solid tumors, melanoma, leukemia, MM, breast, NHL	Sznol and Chen (149), clinicaltrials.gov

*HDAC, histone deacetylase; ADCC, antibody-dependent cellular cytotoxicity; MM, multiple myeloma; NHL, non-Hodgkin’s lymphoma; NSCLC, non-small cell lung cancer*.

Many of these agents are already being tested in conjunction with other immune therapies; especially in combination with ADCC-inducing mAbs that target NK cells to attack tumors. Certainly any new therapy utilizing immunostimulatory mAbs must be carefully evaluated in a stepwise manner to avoid possible adverse side effects, such as autoimmunity (113). However, combination therapies allow clinicians to take advantage of mechanistic synergies that can effectively boost NK cell function using agents that have limited impacts when used alone. An appropriate starting platform combines strategies that boost the immune system and block immune suppression (157), and the hard wiring of NK cells makes them particularly receptive to this strategy. Of particular interest, several phase I clinical trials are currently in progress that combine antibodies designed to block multiple inhibitory immune receptors on NK cells and other leukocytes simultaneously. These include combining the anti-CTLA antibody, ipilimumab with the anti-KIR antibody, IPH2102 (lirilumab) to treat advanced tumors (NCT01750580) and an anti-PD-1 antibody (nivolumab) in combination with either ipilimumab to treat melanoma (NCT01024231) or with lirilumab to treat solid tumors (NCT01714739). Combination therapies may also allow the use of more toxic conventional anti-tumor therapies at lower doses when new therapies are added. Also, patients who have weakened immune function, due to prior radiotherapy or chemotherapy, may particularly benefit from NK cell boosting therapies.

In using these immune therapies, it is important to consider the phenotype of individual patients (158, 159). It is possible that most of these agents can be used together in various combinations or with conventional therapies, depending on the biomarkers present in a particular tumor environment. Altogether, there are several new tools in the medicine cabinet that offer the possibility to improve patient outcomes through boosting NK cell functions. While no one-size-fits-all solution is available to universally improving anti-tumor therapy, proper patient screening should allow the application of personalized combination therapies that harness the beneficial attributes of NK cell-mediated anti-tumor responses to systematically improve overall patient survival.

## Conflict of Interest Statement

Kerry S. Campbell and Adam D. Cohen have consulted for and received funding to study elotuzumab from Bristol-Myers Squibb, which provides partial salary support for Ashley Mentlik James.
